# Living with Transthyretin amyloid cardiomyopathy from a patient perspective

**DOI:** 10.1186/s12872-025-05282-7

**Published:** 2025-11-12

**Authors:** Quan M. Bui, Julia McCain, Larry A. Allen, Eric D. Adler, Cheryl Anderson, Cinnamon Bloss, Borsika A. Rabin, Marcus A. Urey

**Affiliations:** 1 Division of Cardiovascular Medicine, Department of Medicine, University of California, 9452 Medical Center Drive #7411 La Jolla, San Diego, CA 92037-7411 USA; 2 Altman Clinical and Translational Research Institute Dissemination and Implementation Science Center, University of California, La Jolla, San Diego, CA USA; 3 Division of Cardiology, Department of Medicine, University of Colorado School of Medicine, Aurora, CO USA; 4 Herbert Wertheim School of Public Health and Human Longevity Science, University of California, La Jolla, San Diego, CA USA; 5 Center for Empathy and Technology, T. Denny Sanford Institute for Empathy and Compassion, University of California, La Jolla, San Diego, CA USA; 6 Department of Psychiatry, University of California, La Jolla, San Diego, CA USA

**Keywords:** Amyloidosis, Transthyretin, Cardiomyopathy, Quality of life, Patient outcomes, Qualitative research, Implementation science

## Abstract

**Background:**

Transthyretin amyloid cardiomyopathy (ATTR-CM) is a progressive condition for which disease-modifying therapies are increasingly available. However, limited research has explored the patient experience with ATTR-CM and its impact on quality of life.

**Methods:**

In this single-center qualitative study, we conducted semi-structured interviews with ten participants (either with ATTR-CM or at-risk family members). At-risk family members were defined as asymptomatic individuals with a clinically actionable TTR variant that were identified through cascade genetic testing. Interviews were recorded, transcribed, and analyzed using a hybrid deductive and inductive approach. Two independent experts assessed participants’ knowledge of ATTR-CM.

**Results:**

Four exploratory themes emerged regarding the ATTR-CM patient journey. Participants demonstrated moderate to excellent knowledge of disease and an appropriate level of confidence. Diagnostic delays were a major source of frustration and were largely attributed to limited knowledge among non-specialist providers. While these providers often recognized that something was wrong, they struggled with appropriate diagnostic work-up and timely specialist referrals. Patients also noted disparities in ATTR-CM care based on geography, race, and socioeconomic status. Reactions to support groups were mixed, with concerns about re-traumatization being most prominent.

**Conclusions:**

This qualitative study explores aspects of the ATTR-CM patient experience not captured through conventional clinical pathways. Diagnostic delays appeared to be driven by non-specialist provider knowledge gaps and systemic disparities in access to specialized care. These preliminary insights may inform the design of multidisciplinary care models that better reflect patient needs and priorities.

**Supplementary Information:**

The online version contains supplementary material available at 10.1186/s12872-025-05282-7.

## Introduction

Transthyretin amyloid cardiomyopathy (ATTR-CM) has increasingly available disease-modifying treatment options, but delays in diagnosis and barriers to therapy remain common [1, 2]. Several studies have shown that most patients are diagnosed more than six months after symptom onset and typically undergo evaluation by more than two physicians, including cardiologists [3, 4]. These delays are often attributed to the perceived rarity of disease, nonspecific symptoms at presentation, and phenotypic overlap with other cardiac conditions [5, 6]. 

Fortunately, there is growing recognition of ATTR-CM in other common cardiovascular diseases including heart failure with preserved ejection fraction (HFpEF), hypertrophic cardiomyopathy and aortic stenosis [7–9]. Although advances in cardiac scintigraphy have facilitated noninvasive diagnosis, certain genetic variants (i.e. Phe64Leu, Val30Met) may not be reliably detected by bone avid tracers [10]. Ultimately, timely and accurate diagnosis is essential, as prognosis worsens and the efficacy of therapies declines in later disease stages [2, 11]. 

While there has been an appropriate focus on diagnostic medical criteria, there remains a notable gap in qualitative research exploring the lived experience of patients with ATTR-CM [5, 12]. Understanding patient and at-risk family member perspectives is essential to inform implementation of diagnostic and treatment strategies. The objective of this study was to characterize the patient journey and elucidate barriers to diagnosis and management of ATTR-CM through semi-structured interviews.

## Methods

### Design

This single-center qualitative study included patient needs assessment interviews conducted between June 1 and August 30, 2024. A qualitative study design was used to reveal themes that facilitate an in-depth understanding of patient perspectives [13]. The study was overseen by a multidisciplinary research team with expertise in advanced heart failure, cardiac amyloidosis, genetics, genetic counseling, qualitative methodology, and implementation science. The study received appropriate ethical oversight and was approved by the institutional review board.

### Participants

Participants were recruited from the UC San Diego Cardiac Amyloidosis Clinic through two approaches: (1) direct referral from treating heart failure cardiologists (Q.B. and M.U.), and (2) outreach via telephone by the study team to eligible patients and at-risk family members identified during routine clinical care. We used purposive sampling to ensure representation across key demographic characteristics. Although formal demographic data were formally collected at the beginning of each interview, the study team had access to basic demographic data (i.e. race, sex) via the electronic medical record, which informed our sampling strategy. Inclusion criteria included adults (≥18 years) who were either diagnosed with ATTR-CM or were first-degree relatives of affected individuals, English-speaking, and willing to participate in a recorded interview. Exclusion criteria included cognitive impairment that limited ability to consent or participate in interviews. All participants provided informed consent prior to participation in this study.

### Data collection

Interview guides were developed based on discussions with the core research team and were iteratively refined. The interview included various questions organized by three domains (Patient Disease History, Genetic Testing and Counseling, and Health Information Delivery), which were chosen based on prior clinical experience and theoretical frameworks relevant to patient-centered care. Data pertaining to genetic testing were analyzed separately and have since been published in a separate manuscript [14]. The interview guide is included in the Supplement (Supplemental Table 1). In addition to the semi-structured interview questions, participants were asked about demographic characteristics (i.e. self-identified race, highest level of education, occupation, health insurance, etc.). Participants were asked about overall understanding of ATTR-CM and to report on their confidence in their explanation (scale 1–10). Overall understanding (low, moderate, and excellent) was graded based on their explanation (accuracy and depth) of ATTR-CM and by the clinical judgements of amyloid specialists (Q.B. and M.U.). Each case was reviewed jointly, and a mutual conclusion regarding knowledge level was reached. No standardized instruments or predefined criteria were applied. Interviews were conducted and recorded using Zoom by the lead author (Q.B.) who is an expert in the content and discussion topics. No one else was present for the interviews besides Q.B. and the participants. Repeat interviews were not conducted. Participants did not receive monetary compensation for their participation. Field notes were completed after the interviews.

### Analysis

Recorded interviews were transcribed professionally using GoTranscript services and were verified for accuracy by the lead author (Q.B.). All raw data were de-identified, and participants were assigned unique code numbers to ensure confidentiality. Transcripts were not returned to the participants for comments or corrections. We conducted a hybrid thematic analysis, combining deductive and inductive approaches. Initial codes were developed deductively, based on the interview guide and prior clinical experiences related to genetic testing and counseling in cardiomyopathy patients. Codes reflected concepts such as “delays in medical care,” “decision to accept genetic testing,” “risks of genetic testing,” “importance of a genetic counselor,” “informed consent,” etc. However, the analysis remained open to inductive insights that were identified from participant narratives. This approach allowed us to examine anticipated barriers while remaining open to novel perspectives. Our analysis was grounded in post-positivist paradigm, with the aim of identifying shared experiences while acknowledging the influence of the research context. A subset (30%) of the transcripts were coded together by both the lead author (Q.B.) and co-author (B.R.). The remaining transcripts were coded by the lead author (Q.B.). Thematic saturation was assessed iteratively without new themes emerging after coding the seventh transcript. The final three transcripts were used to confirm the stability of the thematic framework. Theoretical sufficiency was used to guide sample size determination. Qualitative data were managed using the qualitative software package, ATLAS.ti v.23.0 (Scientific Software Development GmbH, Berlin, Germany).

## Results

From June to August 2024, we interviewed ten participants (patients with ATTR-CM [*n* = 8] or at-risk family members [*n* = 2]). At-risk family members were defined as asymptomatic individuals with a clinically actionable TTR variant (classified as pathogenic or likely pathogenic) that were identified through cascade genetic testing. Importantly, none of the participants were from the same family. Interviews were recorded, transcribed, and analyzed using a hybrid deductive and inductive approach. Two independent experts assessed participants’ knowledge of ATTR-CM. Interviews lasted between 35 and 65 min. Participants had a median age of 69.5 years (IQR 50–71) and were primarily male (80%), Caucasian (70%) and educated with at least a bachelor’s degree (60%) (Table 1). Diagnosis or identification of a genetic variant associated with ATTR-CM was made between years 2017 and 2024 with the majority (70%) occurring after 2020.

**Table 1:** Baseline participant demographics

	Participants (*n* = 10)
Age, years (IQR)	69.5 (50–71)
Male, *n* (%)	8 (80)
Race, *n* (%)	
Caucasian	7 (70)
Black	2 (20)
Asian	1 (10)
Hispanic	0 (0)
Education, *n* (%)	
High School	1 (10)
Associate	3 (30)
Bachelors	5 (50)
Graduate	1 (10)
Insurance, *n* (%)	
Medicare/Medical	5 (50)
Private	4 (40)
None	1 (10)
Genetic Testing Positive, *n* (%)	7 (70)

### Needs assessment interviews

**Fig. 1:**
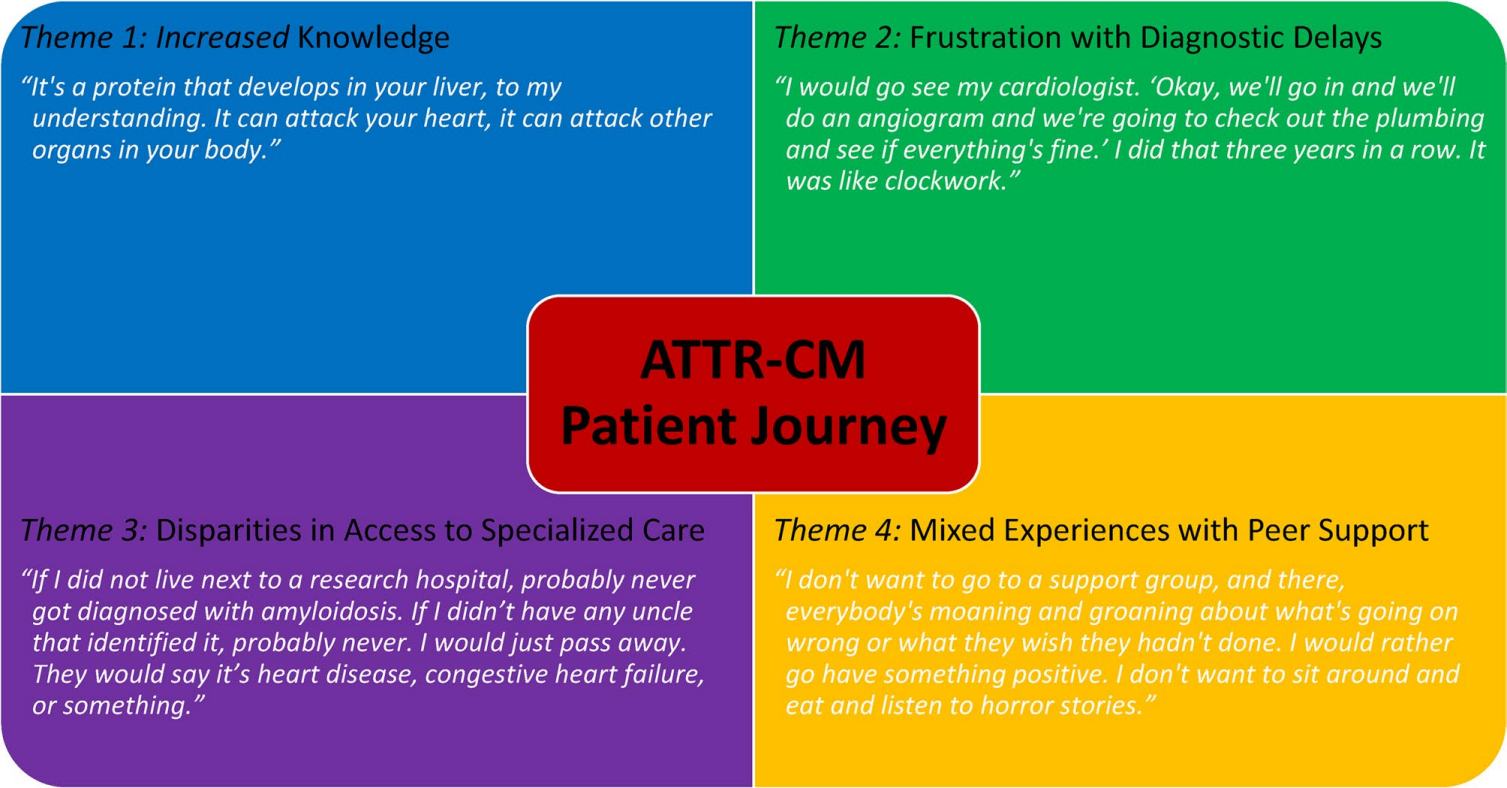
Four Themes Associated with the ATTR-CM Patient Journey

Four preliminary themes emerged from the interviews, characterizing the patient journey with ATTR-CM (Central Illustration): (1) patients demonstrated strong understanding of disease with an appropriate level of confidence; (2) delays in diagnosis were a major source of frustration, largely attributed to limited knowledge among non-specialist providers and insufficient referral to amyloidosis centers; (3) patients identified disparities in care based on geography, race and socioeconomic status; (4) patients expressed mixed feelings towards support groups and online forums with primary concern being re-traumatization (Fig. 1).

### Patient understanding of ATTR-CM

Generally, patients demonstrated a moderate to excellent understanding of ATTR-CM with an appropriate level of confidence. Patients consistently described ATTR-CM as resulting from abnormal proteins that deposit in various organs, leading to dysfunction.

*“I’d say it’s something that causes me to get proteins*,* and I’m not supposed to get too many proteins. It hardens my organs. Then you get sick and die. ” – Participant 10 (hATTR*,* proband) (Understanding: Low; Confidence: 6/10)*‬‬

*“It’s a protein that develops in your liver*,* to my understanding. It can attack your heart*,* it can attack other organs in your body. ” – Participant 1 (wild type (wt) ATTR*,* proband) (Understanding: Moderate; Confidence: 5/10)*‬‬‬‬

*“My amyloidosis is hereditary ATTR. It is a genetically derived disease that mucks up your body’s ability to create proteins. The proteins are created in such a way that they’re misfolded*,* and they become kind of sticky*,* in your body. Where they lodge and accumulate determines where and how you have symptoms. If they accumulate in your heart*,* you can have heart symptoms. If they accumulate in your central nervous system*,* you can have central nervous system involvement. There’s currently no cure for amyloidosis*,* but there are drugs that can silence that genetic component of the amyloidosis mucked up folding process*,* so that symptoms can be sort of suspended in time. Sort of like stopped at the time that you begin treatment. They’re called silencer drugs. That’s like the basics.” – Participant 7 (genotype positive*,* asymptomatic family member) (Understanding: Excellent; Confidence: 8/10)*‬‬

### Delays in diagnosis are common and frustrating

Patients expressed significant frustration with diagnostic delays, which some attributed to non-specialist provider factors. These delays often spanned several years from the onset of initial symptoms, with the longest delay reported being up to 10 years.

*“He came in one day and showed his bicep and it had sagged a little bit. He said*,* ‘Look at my bicep. It’s weird.’ Everyone was like*,* ‘Oh*,* that’s weird.’ Didn’t think anything of it. Then*,* gradually*,* the symptoms got worse over the course of his 50s. I think he died in his early 60s. ” – Participant 7 (genotype positive*,* asymptomatic family member)*‬‬‬‬

*“It didn’t happen overnight. Didn’t happen in three years. It happened over a course of 10 years that I felt crappy for.” – Participant 2 (wtATTR*,* proband)*

Non-specialist providers often recognized that something was amiss, but some struggled with initiation of appropriate diagnostic work-up or timely referral to specialists. For example, patients frequently underwent serial cardiac evaluations, such as stress tests and coronary angiograms, and in some cases, therapeutic procedures that were ultimately unrelated to ATTR-CM.

*“I would go see my cardiologist. ‘Okay*,* we’ll go in and we’ll do an angiogram and we’re going to check out the plumbing and see if everything’s fine.’ I did that three years in a row. It was like clockwork.” – Participant 2 (wtATTR*,* proband)*‬‬

*“I went to the doctor and she had me have an x-ray of my heart. She told me it was enlarged. From then on*,* it just steadily got worse. We had a pacemaker and cardioMEMs and everything else. I was a money man walking around with all this stuff.” – Participant 1 (wtATTR*,* proband)*

A few patients perceived that their non-specialist providers lacked general knowledge and awareness about ATTR-CM. Many expressed surprise and disappointment that even some cardiologists were not well-versed in the diagnosis and management of the disease.

*“My cardiologist at the time told me he was baffled. He didn’t know what to say*,* what to do*,* what to recommend. It was new to him. He had never heard of it before.” – Participant 5 (hATTR*,* proband)*

*“That’s what upset me is the knowledge. Yes*,* patients should have knowledge*,* but I think that every cardiologist should have a complete understanding of what this disease is.” – Participant 2 (wtATTR*,* proband)*

*“I think the biggest problem is not all cardiologists understand*,* or even general practitioners understand amyloidosis is the biggest problem*,* or they don’t think of getting tested for that.” – Participant 9 (wtATTR*,* proband)*

Patients noted that the multisystemic nature of their disease often led to referrals to various specialists. However, these specialists were frequently unaffiliated with amyloidosis centers, and patients observed a lack of coordination and communication among their care teams. Many expressed frustration that non-specialist providers focused primarily on symptoms management without addressing the underlying cause of their condition.

*“I had carpal tunnel. I would go to the orthopedist. I had stenosis of the spine. I would go to another orthopedist. I would go to the cardiologist. I would go to my regular general practitioner because I was tired and fatigued*,* run down. All those symptoms combined should make everyone aware. They don’t communicate. They don’t communicate about the potential disease because of the lack of education or the understanding of how this disease progresses.” – Participant 2 (wtATTR*,* proband)“My last three primary care physicians did not think of amyloid work-up or genetic testing despite my carpal tunnel*,* ankles*,* Achilles heel and biceps tendons issues…I had already been to my primary physician multiple times for amyloidosis-related issues. Here’s your carpal tunnel hand braces. We’re not going to send you to a cardiologist. You tore your Achilles*,* so just stay off that foot. Here’s a boot. They treat symptoms. Over the years*,* they have never asked me to get tested.” – Participant 6 (hATTR*,* family member)*

Patients frequently described feeling compelled to advocate for themselves, despite limited initial knowledge of the disease. Many reported conducting their own research to better understand ATTR-CM and cited medical conferences and symposiums as valuable sources of information. Some patients even took the initiative to educate their providers about ATTR-CM, hoping to improve recognition and reduce diagnostic delays for future patients.

*“I go to some of the symposiums for amyloidosis. Apparently*,* I’ve heard that like central nervous system involvement*,* GI tract stuff*,* that sometimes is earlier*,* and then cardiac is a little bit later.” – Participant 7 (genotype positive*,* asymptomatic family member)*

*“They knew something was wrong*,* but they could not figure it out. I actually sent him (non-specialist provider) a note to let him know that he should check everyone of his patients for amyloidosis. ” – Participant 9 (wtATTR*,* proband)*‬‬

### Limited access to specialists

Patients identified potential disparities in care related to access to ATTR-CM specialists and academic medical centers. They noted that individuals from racially predominant or socioeconomically disadvantaged regions may be disproportionately affected. Amyloidosis centers were perceived as offering not only expert clinical care but also access to advanced diagnostic tools (i.e. genetic testing), novel therapies, research studies, and clinical trials. Patients consistently expressed trust and confidence in the care received at amyloidosis centers.

*“If I did not live next to a research hospital*,* probably never got diagnosed with amyloidosis. If I didn’t have any uncle that identified it*,* probably never. I would just pass away. They would say it’s heart disease*,* congestive heart failure*,* or something.” – Participant 6 (hATTR*,* family member)*

*“In an African American community*,* depending on where you live and access to the medical*,* hospitals*,* doctors*,* things like that. If I lived where I came from (Compton*,* CA)*,* I would never have found this.” – Participant 1 (wtATTR*,* proband)*

*“If you are going to an amyloid specialist and genetic counselor*,* it’s like “We’ve got this.” You happen to stumble on a genetic mutation that they are currently running a study and there’s a treatment for it. If you went to my old doctor*,* he’d be like*,* “I have no idea.” Having access to the right healthcare professional that knows what they’re talking about is key.” – Participant 6 (hATTR*,* family member)*

Rural areas were noted to face unique challenges related to geographic distance from amyloidosis centers. In addition to proximity barriers, patients described a broader cultural skepticism toward the medical system in some rural communities, which may further contribute to delays in seeking care and receiving a timely diagnosis.

*“There’s a culture around that*,* in the farming parts of the upper Midwest in particular. It’s just sort of like*,* you put your head down*,* you don’t talk about it. Your arm could be bleeding*,* like ‘Oh*,* it’s just a flesh wound.’ That’s very much like that*,* ‘Oh*,* we don’t want to burden you with our problems. We’ll just put our head down*,* even though our arm’s falling off*,* we’re just going to keep going*,* no problem.’” – Participant 7 (genotype positive*,* asymptomatic family member)*

*“That was very much their mentality. My sister was like*,* ‘You should go and see someone at Mayo Clinic*,* and see if it’s amyloid-related.’ He was like*,* ‘Oh*,* I don’t know*,* I don’t like doctors.’” – Participant 7 (genotype positive*,* asymptomatic family member)*

### Opinions on peer support groups

Patients reported experiencing anxiety and psychological distress related to their diagnosis of ATTR-CM. As a result, many sought support beyond the medical system, turning to peer support groups, online forums, and social medial platforms. Opinions about these resources were mixed. Some patients found value in validating their symptoms, exploring treatment options, and asking disease-specific medical questions. Notably, these forums remained accessible even to older adults, the demographic most affected by ATTR-CM.

*“I feel I can throw out a question or look back through where people have answered different things. That is significant. I would highly recommend any of your patients to go on that forum and just see what’s the questions that come up… The other day it’s like one of my fingers got stuck for 45 seconds. I’m thinking*,* okay*,* what’s going on here? I go to that forum and I look back and*,* sure enough*,* people had the same issues. Amyloidosis is weird.” – Participant 9 (wtATTR*,* proband)*

*“On one (forum)*,* it’s almost all wild type. You know how people handle different medicine. For some people*,* including me*,* tafamidis didn’t hold up as long as we thought it would. Some of these guys have been on tafamidis for 10 years. It’s obviously slowed it down for them. ” – Participant 9 (wtATTR*,* proband)*

*“I’m on a couple of these forums*,* where we have different questions pop up. I just noticed most of the people are a lot older than I am. I don’t know how many are obviously on that forum. They’re all computer literate because we’re sending stuff back and forth. ” – Participant 9 (wtATTR*,* proband)*‬‬

However, some patients expressed concern that the content on the online forums and support groups was often overtly negative or emotionally overwhelming. As a result, several participants felt these platforms were not beneficial to their well-being and ultimately chose not to engage with them.

*“Honestly*,* I don’t like going to the support groups and stuff because I feel like it’s depressing. It’s like*,* ‘Yes*,* this is what’s going on in my life and this is how much amyloidosis sucks in my life. My husband died.’ I just felt like this isn’t helping me so I decided not to do it anymore. ” – Participant 3 (hATTR*,* family member)*

*“I don’t want to go to a support group*,* and there*,* everybody’s moaning and groaning about what’s going on wrong or what they wish they hadn’t done. I would rather go have something positive. I don’t want to sit around and eat and listen to horror stories. ” – Participant 5 (hATTR*,* proband)*

Patients noted the increasing visibility of ATTR-CM in mainstream media, particularly through television commercials. Overall, they viewed this exposure positively, recognizing its potential to raise awareness among both the public and healthcare providers. However, they also acknowledged the limitations of information presented in these advertisements.

*“Right now*,* I’ve seen a few commercials because you don’t watch Mercedes commercials till you own a Mercedes. I’ve seen amyloidosis commercials. There’s ATTR commercials out there that are starting to come out*,* which is creating a little bit of awareness. Your average family doctor is not even going to think that way. ” – Participant 6 (hATTR*,* family member)*‬‬

*“They started advertising the Pfizer drug on TV and they started saying*,* ‘Look into it because your doctor is probably not diagnosing it.’ They advertised it for a while and I go*,* ‘Oh my God*,* that’s weird.’” – Participant 8 (wtATTR*,* proband)*

*“People haven’t heard about it. You’re starting to see it on TV now*,* medicine and different things about-- but they don’t really say which type of amyloidosis*,* and then that can get confusing.” – Participant 9 (wtATTR*,* proband)*‬‬

## Discussion

In this qualitative study of patients with ATTR-CM and at-risk family members, we identified four exploratory themes that will help incorporate the patient voice into diagnostic and management strategies. First, patients appeared to have an adequate understanding of ATTR-CM and expressed a strong desire for additional disease-specific information. Second, patients reported frustration with diagnostic delays, attributing them to non-specialist providers’ limited knowledge and failure to initiate timely referrals to amyloidosis centers. Third, patients perceived disparities in ATTR-CM care based on proximity to amyloidosis centers, race, and socioeconomic status. Fourth, although support groups were a source of community for some, others expressed concerns about the emotional toll of frequent negative content. Collectively, these findings provide preliminary insights into patient experiences not routinely captured through clinical encounters. These themes should be interpreted in the context of the study’s qualitative design and methodological considerations, which may have influenced how participant narratives were framed and interpreted.

### Delays in diagnosis

Unfortunately, delays in diagnosis are common in ATTR-CM, which affects timely initiation of targeted treatments. Consistent with prior studies of rare diseases, ATTR-CM patients described a prolonged “diagnostic odyssey” marked by uncertainty and emotions distress [12, 15]. Among providers involved in ATTR-CM care, cardiologists are most frequently implicated in misdiagnosis, followed by general practitioners and internists [5]. The most common misdiagnoses included unspecified heart failure (24%), hypertrophic cardiomyopathy (11%) and hypertensive heart disease (5%) [5]. Therefore, the diagnosis of ATTR-CM requires a high index of suspicion among non-specialist providers who are often the first to evaluate these patients [2]. However, advancements in imaging, including cardiac magnetic resonance imaging, and development of novel positron emission tomography radiotracers have improved the ability to distinguish ATTR-CM from phenocopies [6, 16]. In parallel, emerging artificial intelligence tools may support earlier recognition of ATTR-CM by integrating various cardiac diagnostic testing modalities (i.e. electrocardiography, echocardiography, and cardiac scintigraphy) into automated screening algorithms [17–20]. While promising, these tools will require thoughtful implementation into clinical workflows.

### Multidisciplinary collaboration and early referral to specialized centers

A multidisciplinary care model and early referral to amyloidosis centers may help improve diagnosis. In this study, some patients reported that non-specialist providers were able to recognize that something was wrong but occasionally struggled with the initiation of appropriate diagnostic work-up and timely referrals. A few patients underwent repeated cardiac evaluations, including stress tests and angiograms, and even therapeutic procedures that were ultimately unrelated to ATTR-CM. These findings suggest a potential opportunity to improve education amongst non-specialist providers, strengthen partnerships with amyloidosis centers, and lower thresholds for referral. For example, dedicated HFpEF or left ventricular hypertrophy clinics may offer an opportunity to identify more patients with ATTR-CM [21]. The 2023 American College of Cardiology Expert Consensus on Amyloidosis emphasizes a multidisciplinary care model, mirroring management strategies for other systemic cardiac diseases, such as sarcoid, neuromuscular disease and carcinoid syndrome [2]. In a “hub and spoke” program, initial diagnostic testing (i.e. echocardiogram, cardiac scintigraphy, laboratory evaluation for plasma cell disorder) could be performed at community centers before referral to amyloidosis ones. These strategies may be helpful in addressing healthcare challenges with not only underdiagnosis and undertreatment, but also overdiagnosis and overtreatment.

### Disparities in ATTR-CM care

In our study, patients provided preliminary insights into potential disparities in ATTR-CM care, specifically related to access to amyloidosis centers. Community hospitals may lack the infrastructure and specialist support to deliver comprehensive amyloidosis care, including genetic testing. Alexander et al. demonstrated large geographic disparities in amyloidosis-related mortality, suggesting disproportionate underdiagnosis among Black patients [22]. Telehealth may offer a scalable solution to bridge geographic gaps in care and improve access to amyloidosis specialists [2]. These disparities also extend to access to clinical trials, many of which are only available through amyloidosis centers [2]. While three FDA-approved therapies now exist for ATTR-CM (tafamidis, acoramidis, vutrisiran), patients with rare diseases often rely on clinical trial participation to access emerging treatments. These preliminary observations underscore the need to better understand and address disparities in care, with the goal of ensuring equitable access to advances in medicine [23]. 

### Mixed feelings towards peer support groups

Peer support groups are generally viewed as helpful for patients with chronic illnesses, offering both a sense of community an practical strategies for disease management [24, 25]. Prior studies have shown that participation in peer support groups can enhance patient activation, self-efficacy, and self-care behaviors [25]. In our study, some patients described these support groups as validating their symptoms, expanding awareness of various treatment options, and providing answers to disease-specific medical questions. Beyond individual benefits, peer support groups have also been shown to reduce caregiver burden and alleviate strain on healthcare systems [24]. However, peer support groups are not without limitations. Several patients in our study expressed that the tone of online forums and social media discussions often emphasized negative experiences, exacerbating their own psychological distress. Similar concerns have been documented in patients with other chronic conditions, such as inflammatory bowel disease [26, 27]. Patients also described re-traumatization when exposed to other distressing stories from others [26]. Additionally, the unregulated nature of these platforms increases the risk of misinformation, as content is not routinely verified for clinical accuracy [28]. These findings suggest that providers should be mindful of both the benefits and limitations of peer support groups when recommending such resources to patients. In clinical practice, this may involve actively discussing the potential emotional impact of online forums and helping patients identify reliable disease-specific groups. A more intentional approach may help patients obtain the benefits of peer communities while mitigating unintended negative effects.

### Future directions

As targeted therapies for rare diseases continue to expand, understanding the patient experience will be critical to implementation [28]. The 21 st Century Cures Act (2016) and subsequent initiatives by Patient Centered Outcomes Research Institute and National Institutes of Health have emphasized the integration of patient voice and patient-reported outcomes in clinical research [28]. Equally important, incorporating caregiver perspectives will provide a more holistic underdoing of the psychosocial and systemic challenges around diagnosis and treatment in rare diseases [29]. Future studies should use an anchor-based approach to identify outcomes that matter most to ATTR-CM patients, spanning physical, emotional, and quality of life dimensions. In addition, multi-center qualitative studies that include more demographically and socioeconomically diverse populations are needed to improve generalizability and transferability of findings. Mixed-methods research that combines patient and caregiver narratives with quantitative outcomes data may inform the development of truly patient-centered endpoints in future clinical trials [30, 31]. 

### Limitations

There are several limitations to this study. First, the retrospective design introduces the potential for both patient selection and recall biases. Second, the sample included only 10 patients from a single tertiary referral center without follow-up, which limits generalizability. Furthermore, most participants had hereditary ATTR (70%), which further limits generalizability, as the majority of cases in clinical practice are wild type. Although our center draws from a broad geographic region and diverse demographic backgrounds, most participants were Caucasian, well-educated, and demonstrated adequate genetic literacy. As such, findings may not extend to more diverse populations or to other rare diseases lacking available treatments. Third, recruitment through a specialized amyloidosis clinic may have introduced additional bias. Patients referred to tertiary centers may be more engaged with the health system, have greater disease awareness, and encounter fewer structural barriers than those in community health systems. As a result, referral patterns likely shaped the composition of our study participants and the themes identified, potentially leading to an overestimation of patient knowledge and underestimation of systemic barriers encountered in broad clinical contexts. Fourth, the assessments of patient knowledge were based on interviewer and senior author judgements (amyloidosis specialists) without the use of validated tools, which introduces subjectivity and may limit reproducibility. Fifth, caregiver perspectives were not included in this analysis and future research should explore the psychosocial and logistical burdens experienced by caregivers of individuals with ATTR-CM. Sixth, as this was a cross-sectional qualitative study, patient knowledge was assessed after ATTR-CM diagnosis and does not represent knowledge at the time of initial presentation. Seventh, member-checking of interview transcripts was not performed, representing a limitation in qualitative methodology. This may have limited opportunities to validate and refine the identified themes. Finally, the reflexivity of the interviewer, who is an amyloid specialist, may have influenced both participant responses and data interpretation. While this expertise facilitated depth of discussion, it may also have introduced bias in framing questions or interpreting themes.

## Conclusions

In this small, single-center study, patients with ATTR-CM and their at-risk family members provided exploratory insights into the lived experience of this complex disease. Delays in diagnosis appeared to be a significant challenge, often related to non-specialist provider knowledge gaps and inadequate referral practices. Patients also recognized disparities in ATTR-CM care access and expressed mixed views regarding the emotional impact of support groups. These preliminary findings highlight the benefits of a multidisciplinary, patient-centered approach to ATTR-CM care, one that prioritizes timely diagnosis, equitable access to treatment and clinical trials, and integration of the patient voice into research.

## Supplementary Information

Supplementary Material 1.

## Data Availability

The data that support the findings of this study are available from the corresponding author, QB, upon reasonable request.

## Abbreviations

TTR: Transthyretin
CM: Cardiomyopathy
ATTR-CM: Transthyretin amyloid cardiomyopathy
HFpEF: Heart failure with preserved ejection fraction
wtATTR: Wild type transthyretin amyloidosis
hATTR: Hereditary transthyretin amyloidosis
