# Supplementary material for: Living with Transthyretin amyloid cardiomyopathy from a patient perspective
Source: BMC Cardiovasc Disord. 2025 Nov 12;25:802. doi: 10.1186/s12872-025-05282-7 (PMC12613388; doi:10.1186/s12872-025-05282-7)
Supplement: Supplementary file 1 — Supplementary Material 1. [file 12872_2025_5282_MOESM1_ESM.docx]

**Supplemental Table 1: Interview Guide**

PART 1: Patient Disease History

*Next, I will ask you some questions about your heart condition.*

- Can you tell me when you were first diagnosed with your heart condition.
  - What is your understanding of your illness? How would you describe your condition to a stranger?
  - How confident do you feel about your understanding of your heart condition?
  - Are you satisfied with the current work-up and treatment for your heart condition?
  - Are you aware that there are genetic causes of heart disease?
- Has your medical provider ever discussed genetic testing as part of the work-up of your heart condition?
  - *If yes*, please explain what you understand about genetic testing.
    - Describe the process of genetic testing.
    - What is your understanding of the benefits of genetic testing?
    - How might genetic testing be useful in identifying additional family members at risk?
    - What are some of the treatment options available for individuals diagnosed with a genetic heart condition?
    - What are the limitations and risks of genetic testing?
  - *If no*, how interested would you be interested in receiving more information about genetic testing related to your heart condition?
    - What information do you wish you could know?

PART 2: Genetic Testing and Counseling

*Now, I will ask you some detailed questions about genetic testing and counseling.*

- Tell me about your decision-making process for genetic testing.
  - How did (would) you come to your decision?
  - Who helped (would help you) you with making your decision? (*Probe – did you have any family members or friends or clinical care team help?)
  - What information do you wish you had known when making the decision?
  - Were you shown any educational tools or decision aids to help you in the decision-making process?
    - If yes, what were they? How did you feel about them? Did they help you make your decision?
  - Do you wish you had more information? What type?
- How did you feel during the decision-making process?
- What were the positive parts of the decision-making process?
- What were the challenging parts of the decision-making process?
- How did you feel about the explanations you received regarding the risks and benefits of genetic testing? (*Probe – did you feel that you were adequately informed?)
- What are your thoughts on the informed consent process you experienced related to genetic testing?
- Did you make your decision on the spot, or did you take additional time?
- Were there any things that surprised you about the genetic testing process?

*[If patient declined genetic testing]*

- What were the top three reasons that influenced your decision to decline genetic testing?
- How did potential effects on your family influence your decision regarding genetic testing?
- What concerns did you have about potential insurance and workplace discrimination risks related to genetic testing?
- How were your family members approached for clinical screening after you declined genetic testing?

*[If patient received genetic testing]*

- How did your medical provider discuss results of genetic testing with you? In-person visit or over the phone/zoom? Were you satisfied or did you want more information? Did you use the internet/google search?
- How would you describe your medical provider’s understanding of genetics and genetic testing?
- How did your medical provider support communication with your family members regarding genetic testing? Did he or she provide you with a letter to share with them?
- In what ways did your medical provider offer psychosocial support to you and family members? Did you find it sufficient?

*[Genetic counseling]*

- How was a genetic counselor involved in your genetic testing process? Before/after testing or both?
- How did your medical provider approach the topic of referring you to a genetic counselor?
- How did your medical provider explain the role of a genetic counselor?
- What importance did your medical provider place on seeing a genetic counselor, and what benefits were discussed with you?
- Did you end up seeing a genetic counselor?
  - *If yes*, did you find it helpful/useful?
    - How did the information provided by your genetic counselor compare to that provided by your medical provider? Please elaborate.
    - How did your genetic counselor’s interpretation of your genetic testing results compare to that of your medical provider?
    - In what ways did your genetic counselor assist with cascade testing for your family members (if indicated)?
    - How did you feel about the psychosocial support provided by your genetic counselor for you and your family members?
    - Was your visit with the genetic counselor in-person or via telehealth? How did that impact your experience?
    - Do you think having a genetic counselor in the same clinic as your medical provider would be helpful? Please elaborate. (*Probe: would you be more likely to see a genetic counselor?)
- How do you feel about your decision to undergo genetic testing? How did the results and implications of genetic testing compare to your expectations?

PART 3: Health Information Delivery

*Now I’m going to ask you some questions on how you prefer to receive health information.*

- How do you think a decision support tool could assist in decision-making process around genetic testing?
- How do you prefer to receive health information?
  - Online, print, video, verbally (over the phone), person-to-person?
  - Can you give an example of something that was helpful?
    - In what format would you be most likely to read health information?
- How would you feel using an online modular system?
  - Would you feel comfortable? Interested?
- How would you feel watching a 15-minute educational video?
